# Supplementary material for: Recursive Objective Space Exploration (ROSE): A computationally efficient deterministic approach for bi-objective optimization
Source: PLoS One. 2025 Aug 1;20(8):e0327994. doi: 10.1371/journal.pone.0327994 (PMC12316258; doi:10.1371/journal.pone.0327994)
Supplement: S1 File — This file contains supplementary analysis and figures demonstrating the robustness of the ROSE algorithm to different initial seed points in the bi-objective benchmark problem, as well as pseudo-code for the N-dimensional generalization of the ROSE algorithm. (PDF) [file pone.0327994.s001.pdf]

Supplementary Material

S1 Text. Sensitivity of ROSE to the Initial Guess

We investigated the effect of varying the initial guess (seed) on the performance of the ROSE algorithm by conducting six independent runs with different starting points on the bi-objective benchmark problem. ROSE and the baseline NBI/SF approach were compared in each run. All SOOPs were solved with SciPy's trust-constr algorithm. NBI/SF used 98 initial points and a SmartFilter threshold of $\Delta t = 0.01$ to match ROSE's resolution. The resulting Pareto sets, branching patterns, and average solver iterations per significant Pareto point are summarized in Figures 1 and 2.

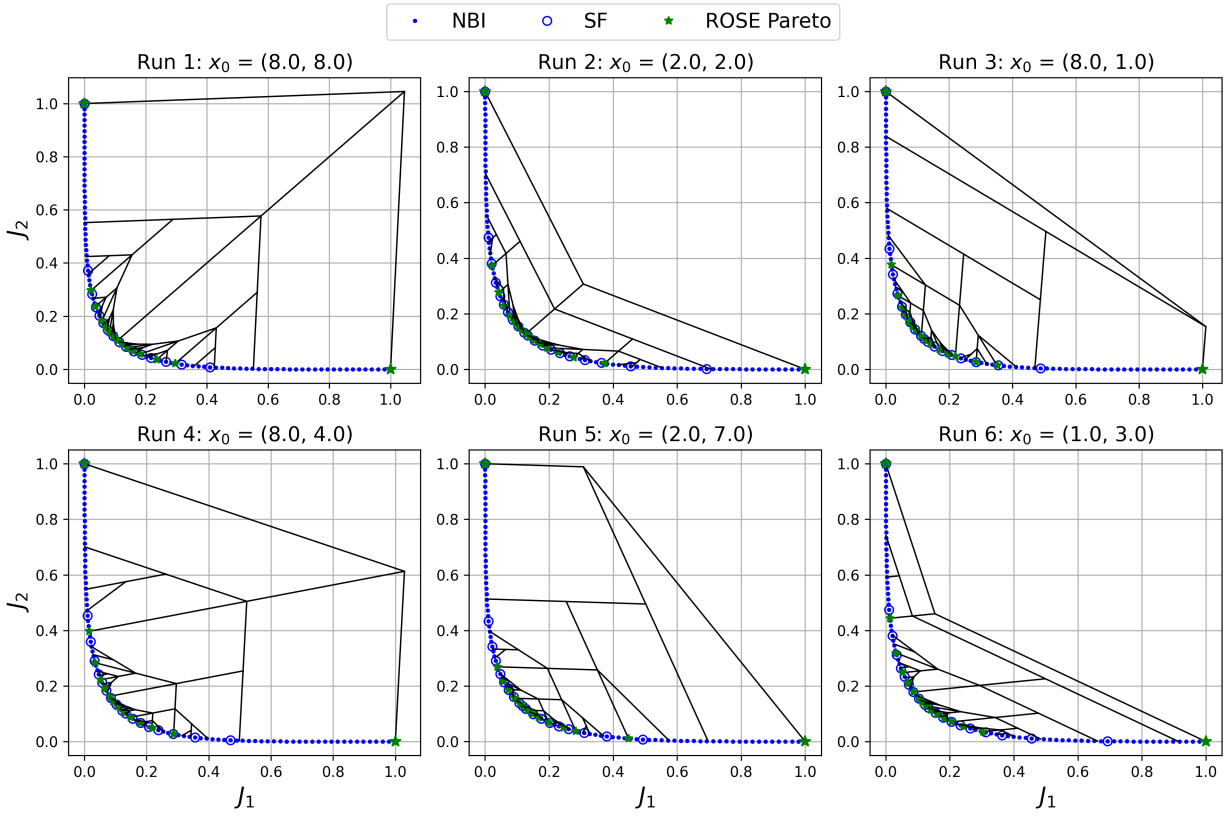

Figure 1: *

Comparison of ROSE and NBI/SF on the bi-objective test problem, initialized from six different seed points x_0. For each run, the branching pattern of ROSE is shown (black lines), along with the Pareto sets identified by ROSE (green stars) and NBI/SF (blue dots, blue circles after filtering).

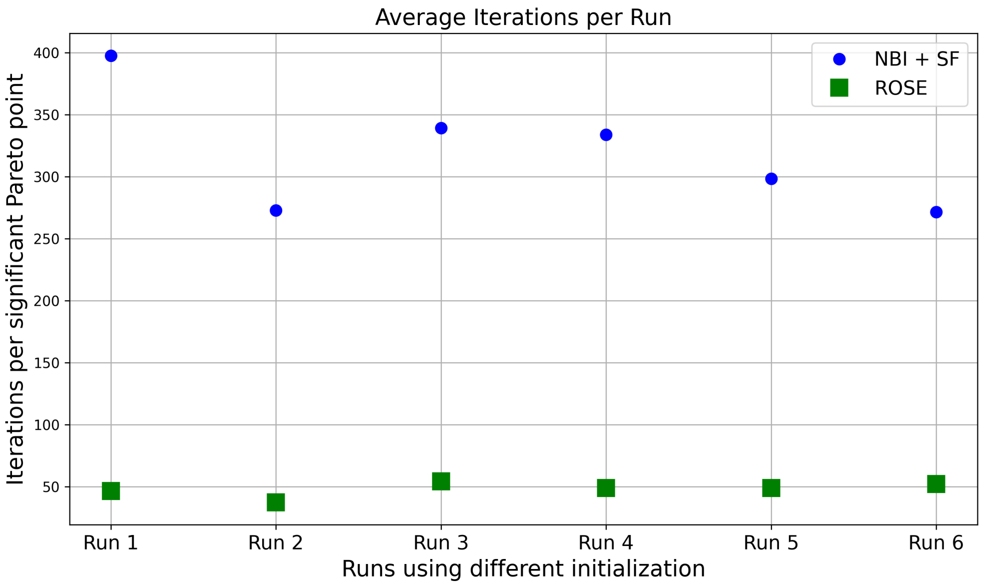

Figure 2: *
Average number of optimization iterations per significant Pareto point for ROSE (green squares) and NBI/SF (blue circles) across six initializations. ROSE is robust to the choice of seed and consistently outperforms NBI/SF in computational efficiency.

S1 Algorithm. Recursive Objective Space Exploration in N-Dimensional Space via Barycentric Subdivision

We provide below the pseudo-code for the general N-dimensional extension of the Recursive Objective Space Exploration (ROSE) algorithm, utilizing barycentric subdivision for recursive partitioning of the objective space.

Algorithm 1 Recursive Objective Space Exploration in N-Dimensional Space via Barycentric Subdivision

Input: Initial guess x_0, and a predefined stopping criterion.
Output: Pareto set S.

Step 1: Initialize the solution set $S = \{\}$.
Step 2: Generate the seed point based on x_0: $(J_1(x_0), J_2(x_0), \ldots, J_N(x_0))$
Step 3: Solve $\min_x\{J_1\}$, $\min_x\{J_2\}$, ..., $\min_x\{J_N\}$ to get anchor points $A_1, A_2, \ldots, A_N$.
Step 4: Start the recursive process with branching node S and the N-dimensional simplex formed by anchor points $A_1 \ldots A_N$.
Construct the bisecting vector $\vec{B}_{A_1,\ldots,A_N}$ for the simplex.
Solve sub-problem $\min_x -|\vec{B}_{A_1,\ldots,A_N}|$ to find Pareto point P_i.
if stopping criterion not met **then**
Add P_i to S
Find node N_i closest to the branch center.
Divide the parent simplex into smaller simplices via barycentric subdivision,
and recursively apply the function to each child simplex with node N_i.
Step 5: When all recursive calls are exited, output the solution set S.
