# Supplementary material for: Recursive Objective Space Exploration (ROSE): A computationally efficient deterministic approach for bi-objective optimization
Source: PLoS One. 2025 Aug 1;20(8):e0327994. doi: 10.1371/journal.pone.0327994 (PMC12316258; doi:10.1371/journal.pone.0327994)
Supplement: Attachment — Submitted filename: ReplyToReviews.pdf [file pone.0327994.s002.pdf]

Manuscript number: PONE-D-25-13070

Title: Recursive Objective Space Exploration (ROSE): A Computationally Efficient Deterministic Approach for Bi-Objective Optimization

Authors: Ihab Hashem, Viviane De Buck, Seppe Seghers, Jan F. M. Van Impe

Response to Reviewers: We thank the Editor and the reviewers for their thorough evaluation and constructive comments. Below, we provide a detailed, point-by-point response to each comment. Reviewer comments are presented in shaded boxes and our replies follow each one.

Authors' Response to Reviewer 1

Comment 1

A) Although an extension to tri-objective problems is proposed, it's not comprehensively benchmarked or validated. Only a small, simple 3-objective problem is tested.

Response:

We thank the reviewer for this observation. We agree that the tri-objective example included in the manuscript is not a formal benchmark. As our focus in this paper is on bi-objective optimization, the three-objective case is presented solely as a conceptual demonstration to illustrate the potential generalization of the recursive strategy to higher-dimensional objective spaces. To clarify this, we added the following sentence at the start of Section 5.3.2:

While this paper focuses primarily on bi-objective optimization, we include here a conceptual extension to three objectives. This section is not intended as a performance benchmark, but as an exploratory demonstration of how the recursive branching scheme can be generalized to higher-dimensional objective spaces.

We also expanded the discussion at the end of this section to highlight specific challenges associated with higher-dimensional extensions, such as spatial bias from simplex-based subdivision and the increasing sensitivity to seed placement. The following paragraph was added:

This reflects the geometric bias introduced by triangle subdivision near the seed. Nevertheless, the recursive branching structure generalizes naturally to higher dimensions. Regarding stopping criteria in multi-objective optimization, possible strategies include direct trade-off comparisons between generated points, centroid-based relevance tests, and information-theoretic measures based on local objective variance Hashem et al. (2017). Two key challenges for future extensions are ensuring uniform exploration of high-dimensional trade-off surfaces and mitigating sensitivity to the initial seed placement, which becomes increasingly critical as the number of objectives increases.

Comment 2

B) There is no formal computational complexity analysis of ROSE (e.g., O(n) or O(log n) behavior), which would help quantify its advantage over NBI/SF more rigorously.

Response:

We agree with the reviewer that formalizing the computational complexity of ROSE strengthens the comparison with NBI/SF. In the revised manuscript, we have now added a complexity analysis at the end of Section 3.1. The following paragraph was added:

Compared to generating the Pareto front using the NBI method followed by Smart Filter (NBI/SF), ROSE utilizes an adaptive strategy. Both approaches have computational costs that scale as $\mathcal{O}(N)$, where N is the number of Pareto points at the chosen resolution. In NBI/SF, a fixed number k of scalarized SOOPs are solved uniformly, resulting in a total cost of $\mathcal{O}(k)$, even though many solutions may be redundant and later filtered out. In contrast, ROSE generates new SOOPs only in regions where the local trade-off exceeds a threshold Δt, yielding $N_{\text{ROSE}} = \mathcal{O}(M)$, where $M \ll k$ in typical cases. In the worst case ($M = k$), both methods solve the same number of SOOPs. Additionally, ROSE reduces the average solver iterations per SOOP by warm-starting each subproblem from a nearby Pareto point. Let I_{NBI} and I_{ROSE} denote the average solver iterations per subproblem for NBI/SF and ROSE, respectively. Since $I_{\text{ROSE}} < I_{\text{NBI}}$ in practice, total costs become $T_{\text{NBI}} = \mathcal{O}(kI_{\text{NBI}})$ versus $T_{\text{ROSE}} = \mathcal{O}(MI_{\text{ROSE}})$, further compounding ROSEs efficiency advantage.

Comment 3

C) The sensitivity of performance to the initial guess (seed) is discussed but not thoroughly investigated with varying objective landscapes or seed placements.

Response:

We thank the reviewer for this point. To address it, we added supplementary material (S1 Text) analyzing ROSEs sensitivity to varying initial seed placements in a bi-objective benchmark problem.

Comment 4

D) A few typos and grammar inconsistencies exist (e.g., lead vs. led, thesef weights), but they dont significantly impact comprehension.

Response:

We thank the reviewer for noting this. The manuscript has been carefully proofread and revised with assistance from a native English speaker.

Comment 5

E) Briefly compare ROSE with vectorization approaches (e.g., MOEA/D, NSGA-II) in discussion, even if not tested. Include pseudo-code in the appendix for the high-dimensional version.

Response:

We thank the reviewer for this suggestion. A brief comparison with population-based vectorization methods has been added to the discussion section of the Conclusion, as follows:

Furthermore, while this work focuses on deterministic scalarization, it is worth briefly contrasting ROSE with population-based vectorization methods such as MOEA/D Zhang and Li (2007) and NSGA-II Deb et al. (2002). These approaches evolve a population of solutions using genetic operators or decomposition strategies. In contrast, ROSE constructs each Pareto point via a directed, recursive search in objective space, offering greater transparency, warm-start potential, and DM-oriented resolution control via

Δt. Although population-based methods remain powerful for many-objective and multimodal problems, benchmarking ROSE against them is an important avenue for future work. Additionally, we included the pseudocode for the generalized high-dimensional version of ROSE in the Supporting Information.

Comment 6

F) Add some recent works that enhance various objectives and applications related to optimizations in your work as below:
https://doi.org/10.1016/j.engappai.2024.108980
https://doi.org/10.1016/j.engappai.2024.108053
https://doi.org/10.1109/JSEN.2024.3477960

Response:

We thank the reviewer for these valuable references. The suggested works have been cited in the Introduction to highlight recent applications of multi-objective optimization.

Authors' Response to Reviewer 2

Comment 1

1) All the figures cannot find in the manuscript.

Response:

We thank the reviewer for noting this issue. All figure references have been carefully reviewed and corrected to ensure that match the figures included in the manuscript.

Comment 2

2) The motivations of developing this algorithm should be clearly given.

Response:

We thank the reviewer for this important comment. To clarify the motivation behind ROSE, we have added the following sentences to the Introduction:

Despite these advances, there remains a pressing need for algorithms that can efficiently allocate computational effort where it matters most, to provide detailed representations of the Pareto front in the regions that are relevant for decision making, while avoiding redundant calculations in uninformative areas. Furthermore, most existing methods lack an intuitive, problem-oriented stopping criterion, often forcing users to rely on arbitrary choices for solution density and termination.

Comment 3

3) Authors should give an intuitive illustration of recursive objective space exploration by a figure.

Response:

We thank the reviewer for this helpful suggestion. We updated Figure 1 to provide a more intuitive explanation of the recursive objective space exploration process employed by ROSE.

Comment 4

4) Authors should conduct the ablation experiments to validate the importance of each main components.

Response:

We thank the reviewer for the suggestion. In response, we added a detailed discussion of the two main components that govern the behavior of ROSE, the initial guess x_0 and the trade-off threshold Δt. Their respective roles are explained more clearly in the Results section, and additional analysis on seed sensitivity has been included in the Supporting Information (S1 Text). The following paragraph was added:

The behavior of the ROSE algorithm is primarily governed by two parameters: the initial guess x_0, which determines the seed point in the objective space, and the trade-off threshold Δt, which sets the resolution of the Pareto set. While x_0 initiates the exploration, the recursive structure shifts focus toward regions of higher trade-offs, making the algorithm relatively robust to the initial seed, as seen across varied runs (see S1 Text). In contrast, Δt directly controls the density of the resulting Pareto front. Smaller values of Δt yield finer approximations in information-rich regions and enable early termination of growth in flat areas, thereby improving efficiency.

Comment 5

5) There are some expensive multi-objective optimization algorithms that have been developed to reduce the computational cost: [1] Liu et al., IEEE Trans. Syst., Man, Cybern. 2021 [2] Lv et al., IEEE/CAA J. Autom. Sin., 2019 [3] Song et al., IEEE Trans. Evol. Comput., 2023

Response:

We thank the reviewer for pointing us to these relevant references. We have now cited these works in the Introduction to acknowledge recent efforts in surrogate-assisted optimization.

Comment 6

6) Please check the paper carefully, there are many typos in the manuscript.

Response:

We thank the reviewer for pointing this out. The manuscript has been carefully proofread and revised to correct typographical and grammatical issues.

References

Deb, K., Pratap, A., Agarwal, S., Meyarivan, T., 2002. A fast and elitist multiobjective genetic algorithm: Nsga-ii. IEEE transactions on evolutionary computation 6, 182–197.

Hashem, I., Telen, D., Nimmegeers, P., Logist, F., Van Impe, J., 2017. A novel algorithm for fast representation of a pareto front with adaptive resolution: Application to multi-objective optimization of a chemical reactor. Computers & Chemical Engineering 106, 544–558.

Zhang, Q., Li, H., 2007. Moea/d: A multiobjective evolutionary algorithm based on decomposition. IEEE Transactions on evolutionary computation 11, 712–731.
